# Traditional Chinese Medicine-Derived Active Ingredient and Formulation Therapy for Glioma: Multi-Target Mechanisms, Drug Delivery Systems, and Advances in Clinical Translational Research

**DOI:** 10.3390/ph19050782

**Published:** 2026-05-16

**Authors:** Xiaoting Shen, Yueling Wang, Yating Lin, Lirong Chen, Hao Wu, Jiaxin Jiang, Lisong Chen, Ying Chen, Desen Li, Wenyi Wang, Shuisheng Wu

**Affiliations:** 1 College of Pharmacy, Fujian University of Traditional Chinese Medicine, Fuzhou 350122, China; 15960615031@163.com (X.S.); 13635269727@163.com (Y.W.); 15880551036@163.com (Y.L.); 18959042801@163.com (L.C.); 18039791170@163.com (H.W.); 18396144318@163.com (J.J.); 18359191896@163.com (L.C.); chenying578119815@163.com (Y.C.); 2 Innovation and Transformation Center, Fujian University of Traditional Chinese Medicine, Fuzhou 350122, China

**Keywords:** glioma, traditional Chinese medicine, multi-target mechanisms, drug delivery systems, clinical translation

## Abstract

Glioma, the most common and aggressive primary brain tumor, presents significant clinical management challenges due to difficulties in blood–brain barrier penetration, high tumor heterogeneity, and susceptibility to drug resistance and recurrence, leading to an extremely poor prognosis. Traditional Chinese Medicine (TCM), particularly its derived active ingredients and herbal formulations, with its advantages of multi-component, multi-target, and holistic regulation, demonstrates significant potential in the comprehensive treatment of this disease. This review systematically outlines the research progress in TCM for combating glioma. Regarding mechanisms of action, active TCM components not only directly inhibit tumors by inducing cell apoptosis but also exert synergistic therapeutic effects via multiple pathways. These include remodeling the immunosuppressive microenvironment, activating novel cell death programs such as ferroptosis and immunogenic cell death, intervening in tumor metabolic reprogramming, and reversing chemotherapy resistance. In terms of overcoming delivery barriers, drug delivery systems represented by nanocarriers, liposomes, and extracellular vesicles, combined with the penetration-enhancing effects of aromatic orifice-opening herbs (a class of TCM medicinals traditionally used to “open the orifices” and awaken the mind, now recognized to transiently enhance BBB permeability), have significantly improved the brain-targeting efficiency and bioavailability of TCM components. For clinical translation, a number of innovative drugs derived from TCM, such as elemene, cinobufagin, and ACT001, are currently under clinical investigation, with initial results showing efficacy in prolonging survival and improving quality of life. In the future, by integrating the analysis of multi-target synergistic mechanisms, promoting the clinical translation of intelligent drug delivery systems, and conducting high-quality clinical research on integrated Chinese and Western medicine, TCM is expected to provide a new generation of integrated treatment strategies for glioma that combines holistic and precision medicine.

## 1. Introduction

Glioma, originating from astrocytes, oligodendrocytes, or their precursor cells, is the most common primary malignant tumor of the central nervous system, accounting for approximately 40–50% of intracranial tumors and exhibiting diverse pathological types [1]. Among them, glioblastoma multiforme (GBM) has the highest malignancy grade [2], classified as a high-grade glioma (WHO grade IV). It constitutes 54% of all gliomas, with an annual incidence of 3.2 per 100,000, and a patient 5-year survival rate of less than 7%, indicating an extremely poor prognosis [3]. In China, the annual incidence of glioma is 5–8 per 100,000, and its 5-year mortality ranks among the highest for systemic tumors [4]. Clinically, patients often present with symptoms such as increased intracranial pressure, focal neurological deficits, and seizures due to the tumor’s mass effect and invasive growth [5]. Currently, the standard treatment for glioma primarily involves maximal safe surgical resection, supplemented by comprehensive approaches including radiotherapy, chemotherapy (e.g., temozolomide, TMZ), targeted therapy (e.g., bevacizumab), and immunotherapy [6]. However, the median survival benefit from the first-line chemotherapeutic agent TMZ is only 14–16 months, and acquired resistance is common [7]. The high genomic heterogeneity of glioma is closely associated with various intratumoral driver alterations (e.g., EGFR amplification, PTEN deletion) [8], leading to low objective response rates (<15%) for single-target drugs (e.g., erlotinib) and difficulty in preventing recurrence [9]. The fundamental therapeutic challenges lie in: the inability to achieve a radical cure through surgery due to infiltrative tumor growth; the difficulty in effective delivery of chemotherapeutic drugs across the blood–brain barrier (BBB); and the propensity for drug resistance with long-term treatment. Therefore, developing novel therapeutic strategies capable of efficiently penetrating the BBB, precisely targeting tumors, and overcoming drug resistance is key to improving patient prognosis.

In this context, the unique value of TCM in comprehensive cancer treatment is increasingly prominent. Its core principles, such as the “holistic view”, “fortifying the body’s vital qi and eliminating pathogenic factors”, and “treatment based on syndrome differentiation” [10,11], align well with the complex pathological mechanisms of glioma involving multiple factors and stages. In TCM, glioma falls under the categories of “headache”, “epileptic syndrome”, and “adverse flow of qi”, among others. Natural products from TCM possess advantages of multi-component, multi-pathway, and multi-target synergistic effects. They demonstrate significant potential in regulating the tumor microenvironment, inducing cell apoptosis, inhibiting invasion and metastasis, reversing chemotherapy resistance, and modulating the body’s immunity, offering new avenues for overcoming existing therapeutic bottlenecks [12,13,14]. Particularly important is that with the integration of cutting-edge disciplines such as nanotechnology and biomaterials, novel TCM drug delivery systems constructed based on nanocarriers, liposomes, and exosomes are effectively addressing challenges associated with traditional TCM formulations, such as low bioavailability and weak BBB penetration, thereby advancing TCM therapy towards precision and high efficiency.

This article aims to systematically review the research progress of TCM against gliomas. First, it outlines the molecular mechanisms by which active TCM components exert anti-tumor effects through multi-target regulation of key signaling pathways, epigenetic modifications, and the immune microenvironment. Second, it provides a focused commentary on the latest achievements of various drug delivery systems in enhancing the brain-targeting delivery efficiency and efficacy of TCM. Finally, it summarizes the current status and challenges in the translation of new TCM drugs that have entered clinical research stages and related advanced technology platforms. Through integrative analysis, this article expects to provide a theoretical basis and forward-looking perspective for elucidating the scientific rationale of TCM against glioma, optimizing targeted delivery strategies, and accelerating the clinical translation of related innovative drugs, thereby promoting TCM to play a more precise and significant role in the multidisciplinary comprehensive treatment system for glioma.

## 2. Multi-Target Mechanisms of Action of Traditional Chinese Medicine in Treating Glioma

Leveraging its core characteristics of “multi-component, multi-target, and holistic regulation”, TCM demonstrates unique advantages in glioma treatment that surpass those of single-target drugs. Its mechanisms of action are not confined to directly killing tumor cells but involve multi-level, multi-pathway synergistic interventions to systematically remodel the tumor and its supportive microenvironment.

### 2.1. Remodeling the Tumor Immune Microenvironment: Transforming “Cold Tumors” into “Hot Tumors”

Glioma, particularly GBM, is a typical immunologically “cold tumor”, characterized by insufficient immune cell infiltration and a strongly immunosuppressive tumor immune microenvironment (TIME), which are key reasons for the failure of immunotherapy [15,16,17]. Recent studies have revealed that various active components of TCM can precisely intervene in the TIME, showing potential to transform “cold” tumors into “hot” tumors—an embodiment of the holistic regulatory advantage of TCM. This concept, while promising, is largely based on the observation of shifted immune cell phenotypes (e.g., M2-to-M1 macrophage repolarization) and altered cytokine profiles in preclinical models. Definitive evidence of a clinically meaningful “cold-to-hot” conversion, characterized by increased T cell infiltration and responsiveness to immunotherapy in patients, remains an area of active investigation. For instance, homogenous magnetic targeting immune vesicles constructed based on arsenic trioxide not only directly kill tumor cells by inducing ferroptosis but, more critically, activate innate and adaptive immunity, systematically reshaping the TIME [18]. Similarly, components such as ginsenosides, chlorogenic acid, and rutin also improve the immunosuppressive state by regulating macrophage M1/M2 polarization, modulating key pathways like EGFR/PI3K/Akt/mTOR, or influencing cytokine networks such as IL-6, IL-10, and TNF-α [19,20,21,22]. Existing research confirms the feasibility of multi-pathway immune regulation by TCM, but most studies remain at the level of phenomenological description and validation of single pathways. Key shortcomings include: First, unclear elucidation of the core direct targets and upstream initiating signals through which TCM components (especially compound formulas) regulate the immune microenvironment. Second, a lack of dynamic, panoramic research on the synergistic changes in various immune cells (e.g., T cells, NK cells, myeloid-derived suppressor cells) and cytokine networks. It should be noted that these findings are predominantly derived from cell and animal models, and their immunomodulatory effects in humans remain to be validated by clinical studies. Future trends require integrating cutting-edge technologies such as single-cell sequencing and spatial transcriptomics to deeply map the fine landscape of the TIME under TCM intervention and clarify the synergistic or antagonistic effects among different components, thereby providing a precise blueprint for designing intelligent TCM formulations with immune-”sensitizing” or -”modulating” functions.

### 2.2. Inducing Novel Forms of Programmed Cell Death: Overcoming Apoptosis Resistance Mechanisms

Traditionally, inducing apoptosis has been a major focus in research on the anti-tumor effects of TCM, with numerous components such as β-elemene, indirubin, shezhi huangling decoction, curcumin, andrographolide, and triptolide confirmed to possess this activity [23,24,25,26,27,28,29,30]. Among these, the apoptosis-inducing effect of elemene has been preliminarily validated in retrospective clinical studies, suggesting potential clinical relevance of this mechanism. However, the widespread apoptosis resistance in glioma cells has driven a shift in research towards novel cell death programs such as ferroptosis and immunogenic cell death (ICD). This shift represents a significant advancement in the field, breaking away from the traditional apoptosis-centered paradigm and revealing the diversity of TCM’s mechanisms of action. Studies indicate that components like dihydroartemisinin, amentoflavone, paeoniflorin, and brucine can induce ferroptosis by regulating key nodes such as ROS, GPX4, and Nrf2 [31,32,33,34], while polyphenols from Erigeron breviscapus can directly trigger ICD, releasing damage-associated molecular patterns (DAMPs) and linking cell death to immune activation [35]. While current research has broken the limitations of traditional apoptosis-inducing therapies and provided new strategies to overcome glioma apoptosis resistance, most studies still merely “validate” known ferroptosis or ICD pathways, lacking innovation. Main issues include: First, a lack of in-depth exploration into the specificity and selectivity of TCM components in inducing novel cell death—why it occurs more readily in tumor cells. Second, the mechanisms of crosstalk between different death modalities (e.g., ferroptosis, apoptosis, autophagy) under TCM intervention remain unclear. At present, the evidence for ferroptosis and ICD is primarily based on in vitro and animal experiments, and their therapeutic value in glioma patients awaits further evaluation in clinical studies. Future research should focus on discovering novel death-inducing mechanisms or targets unique to TCM and systematically investigating the “nodes” where multiple death programs intersect, to develop combined TCM strategies capable of simultaneously activating multiple death pathways and overcoming drug resistance.

### 2.3. Intervening in Tumor Metabolic Reprogramming: Targeting the Vulnerability of the Warburg Effect

Metabolic reprogramming is a key biological characteristic of tumor cells. Glioma cells adjust pathways of glucose metabolism, lipid metabolism, and amino acid metabolism to meet the energy and material demands of their rapid proliferation. Among these, the abnormal activation of glycolysis (the Warburg effect) is a typical manifestation of metabolic reprogramming in glioma, where tumor cells preferentially utilize glycolysis for energy even under aerobic conditions, simultaneously providing intermediates for biosynthesis. Therefore, targeted intervention in tumor metabolic reprogramming, especially inhibiting the abnormally active glycolytic pathway, has become a critical breakthrough in anti-glioma therapy. TCM components such as quercetin, silybin, and toosendanin have been shown to inhibit key glycolytic enzymes or pathways, thereby cutting off the tumor’s energy and biosynthetic supply, reflecting the TCM approach of “treating the root cause” and regulating the internal environment [36,37,38]. Current research mostly remains at the level of demonstrating the overall impact of TCM components on glycolysis or certain metabolite levels, with relatively superficial mechanistic analysis. Significant shortcomings include: First, insufficient identification of the direct enzyme targets or transcriptional regulators through which TCM modulates metabolism. Second, a serious neglect of the critical roles of lipid metabolism and amino acid metabolism in glioma and the potential for TCM intervention. It should be pointed out that the above findings are all derived from preclinical studies, and the translational value and applicability of these mechanisms in clinical settings require further exploration. Future research must integrate technologies such as metabolomics and stable isotope tracing to precisely map the dynamic global metabolic network of tumor cells before and after TCM intervention and identify druggable “metabolic checkpoints”, promoting the translation from “phenomenon” to “target”.

### 2.4. Enhancing Chemosensitivity: Multi-Dimensional Strategies for Reversing Drug Resistance

The sensitivity of tumor cells to chemotherapy drugs directly determines the efficacy of chemotherapy. Sensitive tumor cells exhibit inhibited growth or even apoptosis upon exposure to chemotherapeutic agents, whereas low sensitivity easily leads to drug resistance and treatment failure. In glioma treatment, tumor cells primarily develop resistance to commonly used chemotherapeutic drugs like TMZ and nitrosoureas. TMZ, as a first-line chemotherapeutic agent for glioma, faces resistance mechanisms in some glioma cells, including changes in the methylation status of the MGMT gene promoter, enhanced DNA damage repair capacity, and maintenance of cancer stem cell properties [39]. This issue has become a key bottleneck limiting the effectiveness of glioma chemotherapy. Therefore, finding strategies to reverse chemotherapy resistance and increase tumor cell chemosensitivity is crucial for improving the prognosis of glioma patients. TCM components, leveraging their multi-target regulatory advantages, show significant potential in reversing chemotherapy resistance. Components such as resveratrol and saikosaponin D can effectively reverse TMZ resistance through multiple pathways, including downregulating the expression of the resistance protein MGMT, activating endoplasmic reticulum stress, and regulating the apoptosis/autophagy balance, providing a feasible TCM-based strategy for “chemosensitization” [40,41,42,43,44,45]. Furthermore, 6-gingerol overcomes temozolomide resistance by suppressing epithelial–mesenchymal transition through the PI3K/Akt/β-catenin/c-Myc pathway [46]. The core mechanism by which TCM components enhance chemosensitivity lies in reversing the drug-resistant phenotype of glioma cells, featuring diverse action pathways. These include downregulating resistance-related gene expression and inhibiting DNA damage repair, as well as regulating apoptosis pathways, reversing cancer stem cell properties, and activating endoplasmic reticulum stress. This multi-target regulatory model can effectively address the complex mechanisms of glioma chemotherapy resistance, providing theoretical support for achieving combined “chemotherapy–TCM sensitization” therapy. However, despite clear sensitization effects, current research faces significant translational bottlenecks. First, the vast majority of evidence comes from in vitro cell models, and the complex in vivo microenvironment may significantly affect efficacy, lacking sufficient validation in immunocompetent animal models. Second, research largely focuses on sensitization itself, with a severe lack of exploration into the pharmacokinetic interactions, optimal timing, and dosing regimens for the combined application of TCM and chemotherapeutic drugs, directly hindering clinical translation. However, current research on resistance reversal is mostly concentrated in in vitro cell models and animal experiments, and its clinical translational value still needs to be confirmed by prospective clinical studies. Future research must strengthen preclinical translational study design and actively explore combining TCM sensitizing agents with drug delivery systems to achieve precise co-delivery of synergistic drugs within the tumor.

### 2.5. Promoting Drug Penetration Across the Blood–Brain Barrier: Overcoming the Delivery Bottleneck

The BBB is a major obstacle to drug delivery to the brain. In TCM theory, “aromatic orifice-opening” medicinal substances such as Borneol and Musk have been confirmed by modern research to reversibly open the BBB by modulating tight junction proteins, affecting neurotransmitters like serotonin, and inhibiting efflux pumps (e.g., P-glycoprotein). Their natural origin and unique mechanisms provide valuable tools for brain-targeted delivery [47,48,49]. Furthermore, combining these components with modern drug delivery systems such as nano-liposomes, micelles, and nanoparticles (e.g., Borneol-modified nano-micelles, menthol and casein co-modified 10-hydroxycamptothecin nanoparticles) achieves synergistic effects greater than the sum of their parts (“1 + 1 > 2”) [50,51,52,53]. As natural BBB penetration enhancers, aromatic orifice-opening medicinals are characterized by good biocompatibility and low toxicity. Their mechanisms of action are highly compatible with the physiological regulatory pathways of the BBB. Combining TCM components with novel drug delivery systems like nanocarriers and liposomes can not only further improve BBB penetration efficiency but also enable targeted drug release and reduce off-target toxicity. This field represents a successful example of the intersection between TCM modernization and cutting-edge technology, yet key scientific questions remain unresolved. First, research on the specific molecular switches through which aromatic orifice-opening drugs increase BBB permeability and their safety window (i.e., how to achieve reversible, controllable opening while avoiding side effects like cerebral edema) is insufficient. Second, most current research on delivery systems focuses on increasing brain uptake, with insufficient attention paid to whether, after entering the brain, they can actively target tumor cells and release their payload under specific stimuli. Currently, enhanced BBB penetration effects have primarily been confirmed in animal models, and the safety window and long-term neurotoxicity for human application still require systematic evaluation. The future trend is to develop intelligent, stimuli-responsive brain-targeted drug delivery systems, integrating BBB-opening TCM components, targeting ligands, and therapeutic agents into a unified design, and systematically evaluating their long-term biosafety.

### 2.6. Multi-Dimensional Combined Strategies Synergizing with Standard Therapies

The current standard treatment for glioma is founded on maximal safe surgical resection, combined with concurrent radiotherapy and temozolomide (TMZ) chemotherapy followed by adjuvant TMZ, while recurrent glioblastoma management incorporates bevacizumab and Tumor Treating Fields (TTFields) [7]. However, the median overall survival benefit from standard therapy remains limited, and it is frequently accompanied by adverse effects such as myelosuppression and radiation-induced brain injury. TCM, with its unique advantage of “enhancing efficacy and reducing toxicity”, demonstrates significant value as a bridge in combination with standard therapies. Regarding combination with TMZ chemotherapy, Ligusticum chuanxiong volatile oil can promote the intracellular accumulation of TMZ in glioma cells by downregulating P-glycoprotein [54]; ligustilide combined with TMZ exhibits a synergistic inhibitory effect both in vitro and in vivo with a favorable safety profile [55]; and the essential oil from Ligusticum chuanxiong rhizome maintains a synergistic effect within a multi-target network, especially under hypoxic conditions [56]. Components such as quercetin and tubeimoside I can also reduce MGMT levels by inhibiting the Wnt/β-catenin or EGFR-PI3K/AKT/mTOR pathways, thereby enhancing TMZ sensitivity [57]. A meta-analysis involving 12 trials with a total of 886 patients confirmed that integrated Chinese and Western medicine treatment is significantly superior to Western medicine alone in reducing tumor volume, alleviating gastrointestinal reactions and myelosuppression, and improving the Karnofsky Performance Status [58]. In terms of combination with radiotherapy, natural compounds like curcumin can enhance radiosensitivity by inhibiting the NF-κB pathway [59]; however, low oral bioavailability limits its clinical efficacy [60], underscoring the necessity of developing brain-targeted delivery systems. Furthermore, TCM can alleviate radiation-induced brain injury through its antioxidant and anti-inflammatory effects [61]. In the context of integrating targeted therapy and TTFields, TCM can assist bevacizumab in alleviating peritumoral edema and improve patient tolerance to TTFields by fortifying the body’s vital qi and consolidating the constitution [62]. The Expert Consensus on Clinical Diagnosis and Treatment of Glioma with Integrated Traditional Chinese and Western Medicine explicitly points out the definitive advantages of integrated Chinese and Western medicine in mitigating the adverse effects of radiotherapy, chemotherapy, and TTFields, and in improving quality of life [62]. Current research on combination therapy is still dominated by small-sample, single-center clinical observations, and the optimal timing, dosage ratio, and pharmacokinetic interactions for the combined application of TCM and standard therapies remain to be elucidated. Future efforts should leverage technology platforms such as organoids to systematically screen for optimal combination regimens, thereby facilitating a shift from empirical to precision-based combination strategies.

**Figure 1:**
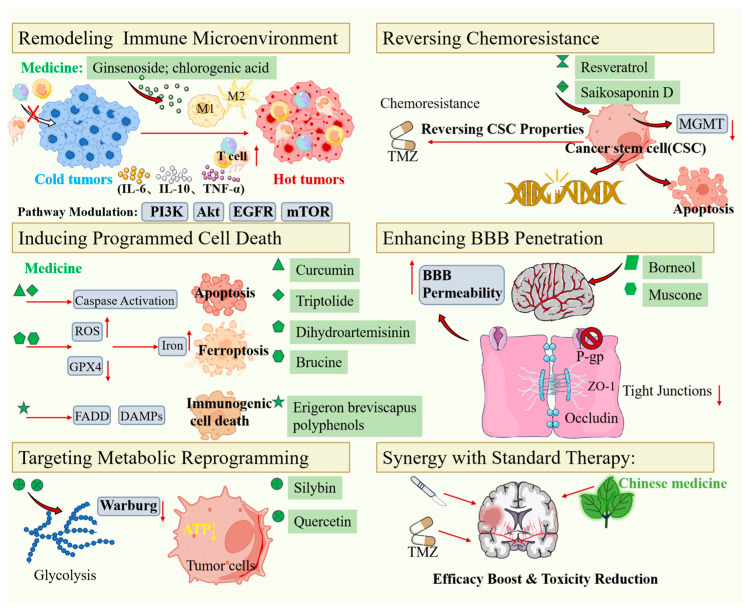
Multi-target mechanisms of action of TCM in treating glioma.

Common multi-target mechanisms of action and representative drugs in Traditional Chinese Medicine for the treatment of glioma are summarized in Table 1 and Figure 1.

## 3. Drug Delivery Systems for Traditional Chinese Medicine in Glioma Treatment

Despite the well-defined anti-glioma pharmacological activities demonstrated by numerous active TCM components (such as celastrol, emodin, and honokiol) in basic research, their clinical translation has long been hampered by inherent shortcomings including poor water solubility, low systemic bioavailability, lack of brain-targeting capability, and difficulty in overcoming the blood–brain barrier (BBB) [63,64,65]. Traditional dosage forms are inadequate for meeting the demands of precision treatment for brain diseases. To overcome this fundamental bottleneck, nano-technology-based drug delivery systems have emerged as a critical solution. These systems, through particle size control, surface engineering, and smart release design, aim to enhance drug enrichment in the brain, prolong circulation time, and reduce off-target toxicity, thereby translating the therapeutic potential of TCM into clinical reality.

### 3.1. Polymeric Nanocarriers: A Platform for Precision Delivery of TCM Active Components

Polymeric nanocarriers, constructed from natural or synthetic polymeric materials such as PLGA, PEG, and chitosan, load TCM active components through encapsulation or covalent conjugation, and can achieve brain-targeted delivery via surface modification with targeting ligands. They have become the most extensively studied and clinically advanced platform in brain-targeted delivery research for TCM active components compared to other nanocarriers, largely due to their chemical versatility and controllable degradation profiles [66,67,68]. A paclitaxel nano-formulation using PEGylated poly(ε-caprolactone) as a carrier not only improved drug solubility but also enhanced anti-glioma efficacy [69]. Co-modification of paclitaxel prodrug self-assembled redox-responsive nanoparticles with borneol and the CGKRK peptide utilizes borneol to facilitate nanoparticle traversal across the BBB, significantly improving anti-glioma efficacy [70]. A brain-targeted delivery system constructed by functionalizing albumin nanoparticles with natural menthol has been proven to be a safe and effective glioma treatment strategy [48]. Notably, although albumin nanoparticles are protein-based carriers—distinct from synthetic polymeric materials—they are frequently categorized under the broader discussion of polymeric nanocarriers in the drug delivery field due to their nanoscale particulate characteristics and analogous formulation principles. In essence, they represent a distinct subclass of natural protein-based nanocarriers with favorable biocompatibility and clinical translational potential. In terms of intelligent stimuli-responsive release, polyethylene glycol-paclitaxel amphiphilic prodrugs linked via boronic ester bonds can self-assemble into micelles and achieve targeted drug release triggered by reactive oxygen species within tumor cells [71]; glutathione-responsive micelles loaded with curcumin not only exhibit good cellular and blood compatibility but also demonstrate superior targeted therapeutic effects against glioma compared to free curcumin [72]. Furthermore, RGD peptide-conjugated solid lipid nanoparticles loaded with asiatic acid also exhibit favorable glioblastoma-targeted delivery capability and therapeutic effects [73]. These studies demonstrate the potential of polymeric nanocarriers to overcome the drug-likeness deficiencies of TCM components, facilitating efficient intracerebral delivery of TCM active substances. However, most polymeric nanocarriers are still in the preclinical research stage; their in vivo targeting efficiency is compromised by factors such as protein corona formation and blood flow shear, resulting in a substantial gap compared to in vitro results. Systematic evaluation of the long-term in vivo accumulation of carrier degradation products and potential immunogenicity is also lacking. Furthermore, the interactions among multiple components in TCM compound formulas further complicate carrier design. Future research should concentrate on the development of carrier materials using clinically approvable excipients, the design of biomimetic multifunctional carriers that integrate the TCM medicinal guide concept, and the establishment of scalable production processes with corresponding quality control systems. Collectively, these efforts will accelerate the shift from paper innovation toward tangible clinical application.

### 3.2. Liposomes: A Biocompatible Delivery System for TCM Active Components

Liposomes are bilayer spheres formed by the self-assembly of amphiphilic molecules. Their unique structure allows them to simultaneously encapsulate hydrophobic TCM active components and hydrophilic chemotherapeutic drugs, making them an ideal carrier for multi-component synergistic therapy [74]. They are one of the classic platforms with the clearest translational prospects, benefiting from established large-scale manufacturing protocols that are currently more mature than those for extracellular vesicles or self-assembled systems. Menthol-modified paclitaxel cationic liposomes can cross the BBB and target tumor stem cells, achieving safe and effective treatment of glioblastoma [75]. Epirubicin and resveratrol co-loaded liposomes, dual-modified with aminophenyl-α-D-mannopyranoside and wheat germ agglutinin, effectively improve drug delivery efficiency across the barrier, demonstrating multifunctional targeting capability and favorable therapeutic effects on brain glioma [76]. Curcumin-loaded nanoliposomes modified with the RDP peptide as a brain-targeted delivery vehicle significantly improve the drug’s tissue targeting, water solubility, and biocompatibility, effectively inhibiting glioma cell growth [77]. Tryptamine-modified liposomal nanoparticles co-loaded with camptothecin and curcumin significantly inhibit glioma cell proliferation and enhance chemo-immunotherapeutic effects compared to unmodified camptothecin [78]. After formulating cinobufagin into PEGylated liposomes, significant advantages over the free drug were observed in terms of hemolysis, cytotoxicity, pharmacokinetics, and antitumor activity, demonstrating pharmacological potential for glioma treatment [79]. However, traditional liposomes still face challenges in systemic circulation, including drug leakage and rapid clearance by the mononuclear phagocyte system, particularly with insufficient encapsulation stability for small-molecule TCM components. Targeting modifications are compromised in vivo by complex factors such as protein corona formation, significantly reducing targeting efficiency. Moreover, the limited tumor tissue penetration capability of liposomes struggles to cope with the highly infiltrative growth characteristics of glioma. Future efforts should focus on developing cell membrane-camouflaged biomimetic liposomes and endogenous stimuli-responsive smart liposomes, utilizing aromatic orifice-opening TCM components to assist in BBB opening, and exploring the combination of liposomes with local drug delivery techniques such as convection-enhanced delivery, enabling this mature dosage form to better serve TCM brain-targeted therapy.

### 3.3. Extracellular Vesicles: Natural Biomimetic Delivery Vehicles for TCM Active Components

Extracellular vesicles (EVs), especially exosomes, as endogenous nanocarriers, represent a highly promising delivery platform for TCM active components due to their inherent biocompatibility, low immunogenicity, intrinsic BBB penetration capability, and intercellular communication functions [80,81]. Hybrid vesicle-based in situ nanovaccines constructed from ginseng-derived vesicles, after intranasal administration, can bypass the BBB, enhance anti-tumor immune responses while inhibiting glycolytic metabolism, and effectively control tumor growth. In this system, ginsenoside Rg3 exerts a brain tumor-targeting effect, while the active component shikonin from Lithospermum erythrorhizon induces immunogenic cell death and inhibits key glycolytic enzymes [82]. Co-loading superparamagnetic iron oxide nanoparticles and curcumin into exosomes modified with the neuropilin-1 targeting peptide on the membrane enables the construction of a glioma-targeted theranostic exosome system. This system can successfully traverse the BBB and accumulate in tumor tissue, achieving tumor imaging and significant anti-tumor effects in vivo [83]. The co-delivery of arsenic trioxide and a photosensitizer using homologous tumor-derived exosomes modified with ginsenoside Rg3 leverages the BBB-penetrating effect of Rg3 and the homologous targeting ability of tumor-derived exosomes, providing a new strategy for the precision treatment of glioma with TCM active components [84]. Another study found that exosomes released by resveratrol-sensitive human glioma cells can enhance the sensitivity of drug-resistant cells to resveratrol, suggesting that exosomes may act as carriers to transmit drug sensitization signals [85]. Following the loading of paclitaxel and doxorubicin into exosomes derived from brain microvascular endothelial cells, the BBB-penetrating capability of this delivery system was validated in a zebrafish brain tumor model [86]. The inherent properties of extracellular vesicles highly align with the TCM concepts of “fortifying the body’s vital qi and eliminating pathogenic factors” and “guiding drugs into the meridians”, yet their clinical translation still faces severe challenges: isolation and purification methods (e.g., ultracentrifugation) suffer from low yields and high batch-to-batch heterogeneity, lacking unified quality control standards; engineering modifications (e.g., drug loading, membrane modification) may disrupt the integrity and function of the native vesicle structure; and safety evaluation of plant-derived exosomes is still in its infancy. Future efforts should advance scalable production processes based on technologies such as tangential flow filtration and microfluidics; establish a multi-tiered quality control system encompassing particle size, markers, and functional potency; and explore the intelligent design of endogenous carriers in conjunction with the TCM channel tropism theory.

### 3.4. TCM Self-Assembled Nanomicelles: A Novel Carrier-Free Delivery System

TCM self-assembled nanocarriers represent a highly original research direction in the field of drug delivery. The core concept involves utilizing the amphiphilic structure of TCM active components themselves or after simple modification to spontaneously assemble them into nanoscale micelles, particles, or fibers through non-covalent interactions. This integrated “drug-as-carrier” design philosophy abandons exogenous synthetic materials, theoretically maximizing drug loading, simplifying preparation processes, and avoiding carrier-related biocompatibility issues [87]. A self-assembled nanocarrier comprising tanshinone IIA and glycyrrhizic acid, coated with engineered exosome membranes, achieved multiple therapeutic effects including enhanced brain drug targeting, BBB penetration, augmented immune responses, and reversal of chemotherapy resistance, providing an innovative therapeutic solution for glioblastoma. An albumin-mediated self-assembled nanodelivery system co-delivering wogonoside and temozolomide triggered potent anti-tumor immune responses by inducing immunogenic cell death, achieving remarkable therapeutic efficacy [88]. A self-assembling peptide nanofiber hydrogel incorporating doxorubicin and curcumin significantly enhanced drug uptake and cytotoxic effects in glioblastoma cells, demonstrating potential for inhibiting tumor recurrence through local administration [89]. However, this strategy highly depends on the intrinsic amphiphilicity of the molecules—a property that the vast majority of TCM active components lack, resulting in a narrow scope of application. Nanostructures maintained by non-covalent bonds are prone to disassembly in complex physiological environments such as blood dilution and protein adsorption, making it difficult to guarantee in vivo stability. Systematic in vivo pharmacokinetic and biodistribution studies are virtually nonexistent. Future research should employ prodrug design and molecular engineering to endow more TCM active components with controllable self-assembly capabilities, introduce orthogonal supramolecular interactions to enhance the integrity of assemblies in vivo, and explore multi-component synergistic self-assembly systems based on the TCM compatibility principle of mutual reinforcement and mutual assistance, thereby propelling this original strategy toward clinical application.

The different types of novel TCM dosage forms mentioned above each have distinct characteristics regarding carrier materials, technological principles, advantages, and application limitations, as detailed in Table 2.

**Figure 2:**
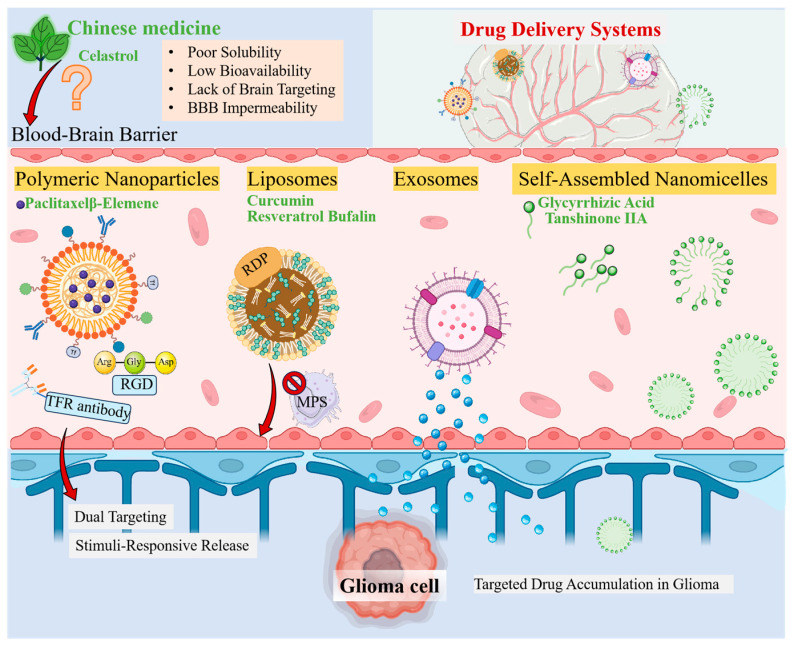
Drug delivery systems for TCM in glioma treatment.

Based on the comparison in Table 2, a clear divergence in clinical readiness emerges among these platforms. Polymeric nanocarriers and liposomes, despite their limitations in targeting precision, still dominate the clinical trial landscape due to scalable production and well-understood safety profiles. In contrast, extracellular vesicles offer superior biocompatibility and the unique ability to cross biological barriers, yet their clinical translation is critically bottlenecked by issues of batch-to-batch reproducibility and the lack of standardized potency assays. The self-assembled nanomicelle system, while conceptually elegant and carrier-free, remains the least mature. Its high dependence on the specific physicochemical properties of TCM molecules, combined with unproven in vivo structural stability, positions it as a high-risk, high-reward strategy far from practical application. Ultimately, the rational choice of delivery system should be a strategic trade-off, tailored to the specific TCM payload’s properties and the targeted therapeutic context, rather than a pursuit of a single ‘best’ platform (Figure 2).

## 4. Clinical Translation and Advanced Technology Platforms for TCM in Anti-Glioma Therapy

The field of TCM-based anti-glioma therapy is currently undergoing a pivotal transition from empirical medicine to evidence-based medicine. Research and development are primarily channeled along two distinct pathways: first, the modern secondary development of established compound formulas (such as Xihuang Wan) or bioactive fractions (such as elemene and cinobufagin) that have demonstrated clinical activity; second, the development of novel drugs from highly potent natural product monomers, exemplified by the parthenolide derivative ACT001. The clinical research progress for these novel agents is summarized in Table 3. Although the aforementioned clinical explorations have revealed encouraging signals, the methodological characteristics of the existing evidence warrant careful interpretation. First, the majority of completed studies are single-center, small-sample, early-phase trials or retrospective analyses (e.g., elemene, Kangliu Wan), with limited sample sizes and a lack of large-scale, multicenter, double-blind randomized controlled designs, which to some extent restricts the generalizability of the results. Second, existing trials typically evaluate TCM as an adjuvant to standard therapy; although this design mirrors clinical reality, it also makes it difficult to precisely quantify the net benefit attributable to TCM alone. Third, regarding endpoints, in addition to overall survival and progression-free survival, neuro-oncologically specific endpoints such as patient-reported outcomes, neurocognitive function, and corticosteroid dosage have not been systematically incorporated. Meanwhile, key molecular markers such as MGMT methylation and IDH mutation status have not been fully utilized for prospective patient stratification, potentially masking the true response of specific subgroups. Finally, the batch-to-batch consistency of compound formulations and the optimal biological dose of single-agent drugs still require further standardization. These limitations do not negate the value of current progress but rather suggest that future efforts should employ biomarker-guided prospective randomized controlled trials, conducted under unified quality control standards, to more accurately evaluate the independent contribution and synergistic value of TCM, thereby providing a higher level of evidence for clinical translation.

Against this backdrop, advanced technology platforms such as organoids, stem cells, and extracellular vesicles provide crucial support for overcoming the aforementioned bottlenecks, propelling TCM research towards precision, personalization, and systematization. Among them, organoid technology, capable of highly simulating patient tumor heterogeneity, tissue architecture, and microenvironment, has become an ideal model for guiding personalized therapy [107,108,109]. It serves three key functions in TCM translation: (1) as a high-throughput screening platform to rapidly evaluate the anti-tumor activity and toxicity of TCM monomers, fractions, or compounds, improving lead compound discovery efficiency (e.g., ACT001’s early development benefited from this); (2) as a personalized drug sensitivity testing tool, enabling “one person, one prescription” precision guidance based on patient-derived organoids; (3) as a mechanistic research model, combining multi-omics technologies in organoids to systematically analyze the synergistic action network of multi-component, multi-target TCM. However, challenges remain: organoid culture and drug sensitivity testing lack unified standards, especially for complex TCM compounds, where methodologies for efficacy evaluation and metabolic analysis are yet to be established.

Furthermore, stem cells (especially mesenchymal stem cells), with their natural tumor tropism, can serve as “living carriers” for targeted delivery of TCM components. Studies show that certain TCM components (e.g., indole alkaloids, gambogamide) can specifically inhibit glioma stem cells, providing excellent candidates for stem cell-based drug delivery systems [110,111]. EVs, due to their low immunogenicity, good biocompatibility, and innate BBB penetration ability, are ideal natural carriers for TCM delivery. Future possibilities include constructing “TCM-EV” hybrid systems that integrate the advantages of plant-derived EVs and synthetic carriers for precise delivery. However, limitations persist: the mechanisms governing stem cell homing and fate control in vivo are not fully understood; and systems for large-scale production, quality control, and biosafety evaluation of engineered EVs urgently need establishment.

The true value of these technologies lies not only in their individual breakthroughs but also in their potential for systematic integration, collectively building a “screening-validation-delivery-therapy” integrated research system. Combining “organoid-AI” could enable virtual screening and efficacy prediction of TCM components. The efficacy of stem cell/EV delivery systems could be pre-validated in patient-derived organoids, forming a closed R&D loop. Furthermore, these platforms provide an interface for synergy between TCM and cutting-edge therapies like immunotherapy, such as evaluating TCM’s role in reversing immunosuppression and enhancing CAR-T efficacy in organoids, or designing engineered EVs that co-deliver TCM and immunomodulatory molecules.

The clinical translation of TCM for anti-glioma therapy must transcend traditional approaches of simple formula modernization or monomer development. Instead, it should construct a new paradigm of “precision integration” and deeply integrate advanced biotechnological platforms like organoids, stem cells, and EVs. Leveraging systems pharmacology and AI to decipher the action networks of compound formulas can push them towards “component-defined, mechanism-describable” component-based TCM. Making brain-targeted delivery system R&D a core part of novel TCM drug design enables “drug-delivery” integrated design. Actively exploring the unique roles of TCM in areas like immune microenvironment modulation and conducting innovative clinical studies combining it with frontier therapies are crucial. In summary, the future of clinical translation for TCM in anti-glioma therapy lies in critically examining existing technological bottlenecks and proactively integrating advanced technologies like organoids, stem cells, and EVs. Only then can TCM be propelled from an adjunctive role to a paradigm shift as a core therapy in glioma treatment, ultimately giving rise to a next-generation cancer treatment system that embodies the holistic regulatory essence of TCM while meeting the requirements of modern precision medicine.

## 5. Conclusions and Future Perspectives

Glioma, especially high-grade glioma, remains a significant therapeutic challenge in neuro-oncology due to its invasive growth, marked tumor heterogeneity, and resistance to apoptosis. Although integrated treatment paradigms centered on surgery, radiotherapy/chemotherapy, and targeted therapy continue to be optimized, overall patient survival rates remain suboptimal, with treatment resistance and recurrence proving difficult to avoid. Within this context, TCM therapy, leveraging its characteristics of multi-component, multi-target, and holistic regulation, offers novel perspectives and strategies for overcoming existing therapeutic bottlenecks. This review has systematically summarized the core mechanisms and cutting-edge progress of TCM against glioma, while also providing a critical appraisal of the limitations in current evidence. By clearly distinguishing between preclinical findings and clinically validated mechanisms, we hope to offer a more nuanced roadmap for future research. At the mechanistic level, TCM not only directly kills tumors by inducing apoptosis but also demonstrates unique advantages in multiple frontier areas, including remodeling the immunosuppressive microenvironment, activating novel cell death programs such as ferroptosis, intervening in tumor metabolic reprogramming, and reversing chemotherapy resistance. At the technological level, novel drug delivery systems represented by polymeric nanocarriers, liposomes, extracellular vesicles, and self-assembled nanomicelles are effectively overcoming the shortcomings of traditional TCM components, such as poor brain-targeting and low bioavailability, propelling treatment towards greater precision. At the clinical translation level, a series of modernized drugs derived from TCM, such as elemene, cinobufagin, and ACT001, have demonstrated potential to improve patient survival and quality of life through clinical trial validation.

However, it is imperative to clearly recognize that the journey of TCM for glioma treatment from basic research to widespread application still faces a series of deep-seated bottlenecks and formidable challenges: (1) The “Black Box” of Scientific Connotation Remains Unfully Opened: The material basis and molecular network of the synergistic effects of multi-components in TCM, especially in compound formulas, are extremely complex. Current research largely remains at the validation of single components or single pathways, lacking a panoramic analysis of the systemic interactions within the “component-target-pathway-phenotype” framework. This severely restricts the in-depth elucidation of its mechanisms of action and the establishment of standardized quality control. (2) The Translational Gap in Delivery Technology: Although novel nano-delivery systems have yielded fruitful results in laboratory research, studies on key translational medicine aspects such as scalable production processes, batch-to-batch stability, long-term in vivo safety, and immunogenicity evaluation are severely lagging. How to translate the “intelligent design” of the laboratory into a “stable product” suitable for clinical use represents the greatest engineering challenge currently. (3) Clinical Research Paradigms Require Upgrading: Currently, TCM in glioma treatment is often positioned as an adjunctive role. Study designs frequently lack biomarker-based precision patient selection, and there is insufficient exploration of innovative clinical protocols combining TCM with cutting-edge modalities like radiotherapy and immunotherapy. This limits the full validation of its efficacy and the elevation of its therapeutic status. (4) Standardization Challenges in Integrating Advanced Technologies: Advanced platforms such as organoids and engineered extracellular vesicles provide powerful tools for TCM research, but they themselves face technological bottlenecks. These include the standardization of organoid culture and drug sensitivity testing, safety control for stem cell therapies, and the large-scale production and quality control of extracellular vesicles. Solving these issues is a prerequisite for these technologies to effectively empower TCM research.

Looking ahead, research and translation of TCM for anti-glioma therapy urgently need to establish a new paradigm of deep interdisciplinary integration, focusing specifically on the following directions: (1) Systematization of Mechanism Research: Integrate systems pharmacology, multi-omics technologies, and artificial intelligence to systematically decipher the multi-dimensional action networks of TCM compound formulas and effective fractions. Promote the evolution from “empirical formulas” to “component-based TCM with clearly defined mechanisms”. (2) Precision in Dosage Form R&D: Prioritize the development of brain-targeted delivery systems (especially bio-derived carriers like extracellular vesicles) as a core component of innovative TCM new drug design. Achieve integrated “drug-delivery” innovation and establish a complete, supportive translational research system. (3) Frontier-oriented Clinical Validation: Proactively design and conduct prospective clinical trials based on biomarker stratification, exploring combinations of TCM with radiotherapy/chemotherapy, immunotherapy, Tumor Treating Fields, etc. Simultaneously, utilize patient-derived organoid models for personalized drug sensitivity prediction, exploring a new precision treatment model of “clinic-organoid-clinic”. (4) Standardization of Technology Platforms: Promote the establishment of high-quality, standardized glioma organoid biobanks and efficacy evaluation platforms. Formulate industry standards for the production and quality control of engineered biological carriers (stem cells, extracellular vesicles), laying a solid foundation for the reliable application of these technologies.

In summary, TCM has demonstrated irreplaceable potential and unique scientific value in the comprehensive treatment of glioma. To achieve the systematic clinical translation of TCM for anti-glioma therapy, concerted efforts must focus on optimizing its large-scale preparation processes and long-term safety evaluation systems, actively promoting the conduct of high-quality, multi-center clinical trials, and clarifying synergistic regimens between TCM and existing modalities such as radiotherapy, chemotherapy, and immunotherapy. This will facilitate the construction of a truly integrated glioma treatment model that merges the strengths of Chinese and Western medicine. On this basis, TCM holds the promise of ultimately giving rise to a next-generation integrated therapeutic strategy. This strategy would both inherit the holistic regulatory philosophy and possess precision treatment characteristics, capable of simultaneously modulating the tumor microenvironment and directly inhibiting tumor growth. It would thereby open up new, effective pathways for improving the prognosis of this formidable clinical challenge that is glioma. Beyond TCM-based strategies, the glioblastoma treatment landscape in 2026 is increasingly shaped by personalized medicine, with advances including gene therapy, biomarker-driven “5G” platform trials, ultrasound-mediated BBB opening, oncolytic virus immunotherapies, and metabolic interventions such as methionine restriction with steroids [10,112,113]. These developments, together with TCM-derived therapies, are fostering a new era of integrated and precision-oriented treatment for glioblastoma.

## Figures and Tables

**Table 1:** Multi-Target Mechanisms of Action and Representative Drugs in Traditional Chinese Medicine for Glioma Treatment.

Action Mode	Summary Mechanism of Action	Related TCM Components/Drugs	References
Remodeling the tumor immune microenvironment	Transforming “cold tumors” into “hot tumors”, promoting macrophage polarization to the M1 phenotype, enhancing T cell infiltration and activation, regulating inflammatory cytokine expression.	Ginsenosides, chlorogenic acid, rutin, ginsenoside Rg3 composite liposomes	[15,16,17,18,19,20,21,22]
Inducing apoptosis	Activating Caspase pathways, regulating the Bax/Bcl-2 ratio, inducing mitochondrial pathway or death receptor pathway apoptosis.	β-elemene, curcumin, tubeimoside I, quercetin, andrographolide, triptolide, indirubin, shezhi huangling decoction	[23,24,25,26,27,28,29,30]
Inducing ferroptosis	Inhibiting GPX4, promoting ROS accumulation and lipid peroxidation, increasing intracellular iron ion levels, activating ferroptosis-related pathways.	Dihydroartemisinin, amentoflavone, paeoniflorin, brucine	[31,32,33,34]
Inducing ICD	Directly triggering FADD protein, releasing DAMPs, activating anti-tumor immune responses.	Erigeron breviscapus polyphenols	[35]
Intervening in tumor metabolic reprogramming (Inhibiting glycolysis)	Inhibiting key glycolytic enzymes (e.g., HK, PKM2), reducing ATP production, inducing energy stress and autophagy/apoptosis.	Silybin, quercetin	[36,37,38]
Enhancing chemotherapy drug sensitivity	Downregulating MGMT expression, inhibiting DNA repair, enhancing endoplasmic reticulum stress, reversing cancer stem cell properties, synergistically inducing apoptosis.	Resveratrol, saikosaponin D, β-Caryophyllene oxide, honokiol, neobavaisoflavone, 6-gingerol	[39,40,41,42,43,44,45,46]
Promoting drug penetration across the Blood–Brain Barrier	Inhibiting P-glycoprotein efflux pumps, downregulating tight junction proteins (ZO-1, occludin), modulating NO levels, enhancing nanocarrier penetration capability.	Borneol, muscone, menthol, gold nanoparticles, casein-modified nanoparticles	[47,48,49,50,51,52,53]
Synergistic combination with standard-of-care therapy to enhance efficacy and reduce toxicity	Enhancing TMZ sensitivity, inhibiting P-gp-mediated drug efflux, enhancing radiosensitivity, reducing adverse effects of chemoradiotherapy, and improving performance status and quality of life	Chuanxiong essential oil, ligustilide, Chuanxiong * rhizome essential oil, quercetin, tubeimoside I, curcumin	[54,55,56,57,58,59,60,61,62]

* Chuanxiong (*Ligusticum chuanxiong hort.*) is a plant whose root has been used in Traditional Chinese Medicine for centuries.

**Table 2:** Characteristics of Different Dosage Forms in the Treatment of Glioma.

Dosage Form Classification	Core Carrier/Technical Principle	Example(s)	Payload (TCM)	Mechanism of Action/Advantages	Limitations
Polymeric nanocarriers	Natural/synthetic polymeric materials (PLGA, PEG, chitosan), surface-modified with targeting ligands (transferrin receptor antibodies, RGD peptides).	PEGylated polycaprolactone nanoparticles loaded with paclitaxel; Borneol + CGKRK peptide-modified redox-responsive paclitaxel nanoparticles; Menthol-modified albumin nanoparticles.	Paclitaxel [48,69,70]	Good biocompatibility, controllable release; Targets BBB and tumor cells; Improves drug solubility; Responsive to tumor microenvironment (pH, ROS) for drug release.	Mostly in preclinical research; Large-scale production processes need optimization.
Liposomes	Amphiphilic phospholipid bilayer structure, surface-modified with targeting groups (mannopyranoside, wheat germ agglutinin, RDP peptide).	Menthol-modified cationic paclitaxel liposomes; aminophenyl-α-D-mannopyranoside-modified epirubicin + resveratrol liposomes; PEGylated cinobufagin liposomes.	Paclitaxel [75]; Epirubicin + Resveratrol [76]; Cinobufagin [79]	Excellent biocompatibility, easy fusion with cell membranes; reduces clearance by mononuclear phagocyte system; co-delivery of multiple drugs; reduces drug toxicity.	Insufficient in vivo stability; prone to drug leakage.
Extracellular Vesicles	Natural nanoscale vesicles (exosomes, microvesicles), drug-loaded and modified with targeting peptides (e.g., neuropilin-1 targeting peptide).	Ginseng vesicle hybrid nanovaccine; superparamagnetic iron oxide + curcumin-loaded exosomes; brain microvascular endothelial cell-derived exosomes loaded with PTX + doxorubicin.	Ginsenoside Rg3 + Shikonin [82]; Curcumin [83]; Paclitaxel + Doxorubicin [86]	Innate BBB penetration capability; low immunogenicity, high targeting specificity; synergy between carrier function and drug efficacy; can bypass BBB via intranasal delivery.	Low preparation yield; lack of quality control standards.
Self-assembled nanomicelles	Self-assembly via non-covalent bonds utilizing the inherent amphiphilic structure of TCM active components (e.g., hydrophilic glycyrrhizic acid sugar chain + hydrophobic aglycone).	Tanshinone IIA-Glycyrrhizic acid self-assembled nanocarrier (exosome membrane-coated); albumin-mediated wogonoside + TMZ self-assembled nanoparticles; doxorubicin-curcumin self-assembled peptide nanofiber hydrogel.	Tanshinone IIA [87]; Wogonoside [88]; Curcumin [89]	Carrier-free, simplifies preparation; strong targeting, penetrates BBB; synergistic chemotherapy and immunotherapy; overcomes drug resistance.	Only applicable to specific amphiphilic TCM components; stability significantly affected by environmental factors.

**Table 3:** Progress in Clinical Research of Novel TCM Drugs for Glioma Treatment.

Drug	Component/ Source	Mechanism of Action	Clinical Research Progress	Stage	References
Elemene Injection	Extract of Curcuma wenyujin (β-elemene)	Induces apoptosis, inhibits angiogenesis, promotes cellular senescence	Combined with TMZ in GBM patients extended OS to 21 months, PFS reached 11 months; Postoperative adjuvant therapy improved immune function and prolonged survival.	Approved (national category II anticancer drug)/phase IV clinical trial	[23,90,91,92,93]
Cinobufagin	Extract of dried toad skin	Inhibits PI3K/AKT/4EBP1 and BAX/caspase pathways, induces apoptosis	Combined with TMZ showed superior efficacy to TMZ monotherapy in malignant glioma and improved patient immune function.	Phase II clinical trial/post-marketing evaluation	[94,95]
ACT001	Parthenolide derivative	Inhibits NF-κB and STAT3 pathways, targets AEBP1/PI3K/AKT signaling	Monotherapy achieved CR in recurrent GBM patients; Designated as a “Breakthrough Therapy” by China’s CDE.	Phase III clinical trial	[96,97,98]
Xihuang Wan	Calculus Bovis, Olibanum, Myrrha, Moschus	Promotes glioma cell pyroptosis, inhibits CD133/EGFR/Akt/mTOR signaling	A phase III trial is underway (Registration No. ChiCTR2300071982) to evaluate its efficacy combined with targeted drugs for recurrent high-grade glioma.	Phase III clinical trial ongoing	[99,100]
Kangliu Wan	18-herb compound formula (includes Hedyotis diffusa, etc.)	Inhibits tumor cell proliferation	Combined with standard Western therapy for malignant glioma resulted in a 1-year survival rate of 97.92% and a median survival of 21.13 months.	Phase II clinical trial/retrospective study	[101]
Paclitaxel	Extract of Taxus (semi-synthetic)	Induces DNA damage and apoptosis	Combined with carboplatin showed anti-glioma activity in preclinical models; A new strategy combining it with Low-Intensity Pulsed Ultrasound (LIPU) to open the BBB preliminarily showed safety and potential to prolong survival.	Phase I completed/phase II ongoing	[102,103]
Chlorogenic Acid	Extract of plants from Caprifoliaceae family	Inhibits Bcr-Abl kinase, promotes macrophage M2 → M1 repolarization	Phase I trial showed good tolerance, MTD 5.5 mg/kg; 52.2% of patients had stable disease, median OS 11.3 months; Phase II/III multicenter studies are ongoing.	Phase I completed/phase II/III ongoing	[104,105,106]

## Data Availability

No new data were created or analyzed in this study. Data sharing is not applicable.

## Abbreviations

The following abbreviations are used in this manuscript:

TCM	Traditional Chinese Medicine
GBM	Glioblastoma multiforme
TMZ	Temozolomide
BBB	Blood–brain barrier
TIME	Tumor immune microenvironment
ICD	Immunogenic cell death
DAMPs	Damage-associated molecular patterns
GMP	Good Manufacturing Practice
EVs	Extracellular vesicles
TTFields	Tumor Treating Fields
